# Quantitative Study of Volumetric Changes of Cerebellum in Male Adult Rat Following Lithium Administration

**DOI:** 10.5812/ijhrba.4187

**Published:** 2012-07-25

**Authors:** Zahra Heidari, Hamidreza Mahmoudzadeh-Sagheb

**Affiliations:** 1 Department of Histology, School of of Medicine, Zahedan University of Medical Sciences, Zahedan, IR Iran

**Keywords:** Lithium, Cerebellum, Bipolar Disorder

## Abstract

**Background:**

Lithium is a drug for treatment of bipolar disorder by correcting mania and reducing depressive mood swings.

**Objectives:**

In this study, effects of Lithium on volumetric parameters of cerebellum were investigated using stereological methods.

**Materials and Methods:**

In this experimental study, 20 sexually mature wistar male rats were selected and divided in two groups randomly (n = 10). Administration and control groups received continuously 0.1 percent Lithium carbonate solution and distilled water respectively with drinking water during a period of 12 weeks. Rat’s cerebellum excised and fixed in modified Lillie’s solution. Then tissues were dehydrated, cleared, and embedded in paraplast in random orientation, and exhaustively sectioned. Ten to twelve sections of ~ 5μm were sampled and stained from each cerebellum by systematic uniform random sampling. The whole section image projected on the table, and using Cavalier’s principle point-counting was employed to estimate the volumetric parameters. Data analyzed by nonparametric statistical test of Mann-Whitney, and differences between groups less than 0.05 considered significant.

**Results:**

There were no significant difference in terms of total volume but gray matter volume of cerebellum increased and white matter decreased in administration group significantly (P < 0.05).

**Conclusions:**

Administration of 0.1% Lithium carbonate for a period of 12 weeks can affect cerebellar gray and white matters in rat.

## 1. Background

Lithium is a mood-stabilizer drug that is used to treat manic depressive and mixed episodes of bipolar mood disorders (1-3). Therapeutic effects of Lithium in patients with chronic and recurrent manic were described in 1949 by Cade. For the past five decades, since its introduction, Lithium has been prescribed as the first line drug in managing bipolar mood disorder that is an efficient drug in controlling manic and depressive crises in bipolar patients with an anti-suicide effect. Nevertheless, toxic reactions are common and are correlated to the serum drug concentration (4, 5). Cerebellar toxicity has also been recognized as an uncommon but potentially irreversible adverse effect on sequence of Lithium therapy (3, 6). Neuromuscular manifestations include proximal muscle weakness, rhabdomyolysis, a myasthenia gravis-like syndrome, and axonal neuropathy (3). Experimental trials demonstrated that Lithium uptake by central nervous system (CNS) is not uniform; its concentration might be high in the brain while it is at therapeutic levels in the plasma. These data suggest that the presence of high serum Lithium concentrations to emerge neurotoxic effects is not mandatory and that Lithium’s neurotoxicity mechanism seems to be multi-factorial. A likely association between Lithium toxicity and cerebellar degeneration has been suggested by neuropath logical studies (6). Sassi et al. using magnetic resonance imaging (MRI) and spectroscopy showed that total gray matter volumes were significantly increased in Lithium-treated patients compared to untreated patients and healthy individuals and concluded it might possibly reflect neurotrophic effects of Lithium (7). Recent studies also have shown unexpected neuroprotective effects in a variety of animal models of neurodegenerative diseases (1). Moore et al. showed that after 4 weeks of chronic Lithium treatment in bipolar patients, gray matter volume of brain increased significantly probably because of neuroprotective effects of Lithium (8). Another study by Chen et al. showed that Lithium has robustly up regulated concentration of cyto protective protein bcl-2 in rodent brains and in human neuronal cells (9, 10). Other studies have recently provided evidence of Lithium-induced increases in gray matter volumes and N-acetyl- aspartate level. N-acetyl- aspartate believed to be localized mainly in dendrites rather than cell body and is a putative marker of neuronal viability and function (8). On the other hand, Licht et al. after a period of 30 weeks in cerebellum and neocortex of rats by using stereological methods reported that there were no statistically significant differences between Lithium treated rats with or without haloperidol and control rats (11, 12). Sassi et al. using MRI measured gray matter volume at right and left anterior and posterior cingulated cortices. These images revealed a volume reduction in left anterior cingulated area in untreated bipolar patients compared to controls but there were no statistically differences between Lithium-treated patients and controls (13). Our previous study showed that there were no significant differences in terms of total gray and white matter volumes of cerebrum between control and Lithium administrated groups after a period of 12 weeks. We concluded that Lithium carbonate 0.1% might not affect volumetric parameters and macroscopic structure of cerebrum in rats (14). In present study, we decided to study the effects of long-term Lithium therapy on volumetric parameters of rat cerebellum using stereological methods.

## 2. Objectives

In this study, effects of Lithium on volumetric parameters of cerebellum were investigated using stereological methods.

## 3. Materials and Methods

In this experimental study, 20 male Wistar rats weighing 250-300 g were selected from animal house of Research center of Zahedan University of Medical Sciences (Iran). There was a constant cycle of 12 h light and 12 h darkness; the temperature was maintained at 22 ± 2 ºC, and rats were permitted to access freely to standard laboratory diet and water ad libitum. After 2 weeks of acclimatization, they were divided randomly into control and experimental groups (n = 10); former group received distilled water and the latter received 0.1% (w/v) Lithium carbonate solution through drinking water for a period of 12 weeks. Then the rats were anaesthetized and their cerebellum were excised and fixed in modified Lillie’s solution for 72 hours at room temperature. The cerebellums were dehydrated, cleared, and embedded in paraplast in random orientation, and then exhaustively were sectioned. Ten to twelve sections of ~ 5 μm were sampled from each cerebellum by systematic uniform random sampling. Each of these sections was stained with hematoxylin and eosin and mounted. In order to project whole section image on the screen, a modified slide projector was used (final magnification 24). Point counting using Cavalier’s principle was employed to estimate the volume of cerebellum using formula:

**Figure:**
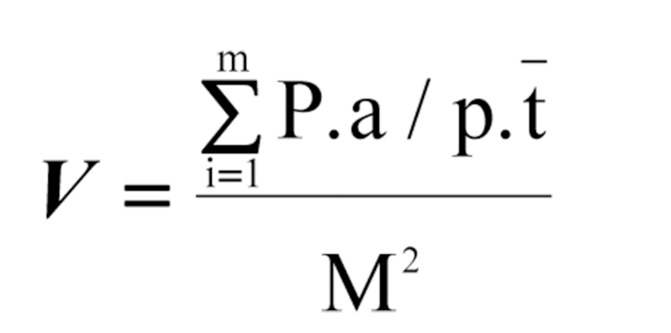


Where ΣP stands for the number of points hitting the object transects, a/p for the area associated with each point in grid, t¯ for mean distance between sections and, M for linear magnification of image. Coefficient error of point counting was calculated as described previously (15). All stereological procedures were carried out by the same person who was blind about experimental status of animals. Data analysis was performed by nonparametric statistical test of Mann-Whitney, and differences between groups less than 0.05 considered significant. All animals received human care, as outlined in the guide for the care and use of laboratory animals. This study was approved by Ethical Committee of Zahedan University of Medical Sciences (Zahedan, Iran).

## 4. Results

Total cerebellar, gray matter, and white matter volumes, and gray/white matter ratio in rats of Lithium-treated and control groups are shown in *Table 1*. Total cerebellar volume in Lithium-treated group increased 3% compared to control group, but this difference was not statistically significant (P > 0.05). Gray matter volume increased 15% in Lithium-treated group compared to control group with a statistically significant difference between two groups (P < 0.001). White matter volume decreased 19% compared to control group that was statistically significant (P < 0.01). Gray/white matter ratio increased 43% in Lithium-treated group compared to control group that showed a statistically significant difference between two groups (P < 0.001) (*Table 2*).

**Table 1.** Mean Values of Total Cerebellar Volume, Gray Matter Volume, White Matter Volume, and Gray/White Matter Ratio in Lithium-Treated and Control Groups

	TCV ^a^	GMV ^a^	WMV ^a^	G/W ^a^
**Control**				
C1 ^a^	245.00	162.00	83.00	1.95
C2	239.00	157.00	82.00	1.91
C3	229.00	147.00	82.00	1.79
C4	260.00	80.00	80.00	2.25
C5	252.00	67.00	85.00	.96
C6	231.00	52.00	79.00	1.92
C7	225.00	43.00	82.00	1.74
C9	226.00	43.00	83.00	1.72
C10	241.00	59.00	82.00	1.94
**Lithium-Treated**				
L1 ^a^	256.00	83.00	73.00	2.51
L2	262.00	79.00	83.00	2.15
L3	232.00	65.00	67.00	2.46
L4	252.00	90.00	62.00	3.06
L5	239.00	78.00	61.00	2.92
L6	243.00	180.00	63.00	2.86
L7	239.00	82.00	57.00	3.19
L8	245.00	176.00	69.00	2.55
L9	265.00	95.00	70.00	2.78
L10	230.00	70.00	60.00	2.83

^a^Abbreviations: C, control; GMV, gray matter volume; G/W, gray/white matter ratio; L, lithium treated; TCV, total cerebellar volume; WMV, white matter volume

**Table 2.** Comparison of Total Volume of The Cerebellum, Gray Matter Volume, White Matter Volume of The Cerebellum, and Gray to White Matter Ratio Between Lithium-Treated and Control Groups

	TCV ^a^, mm^3^	GMV ^a^, mm^3^	WMV ^a^, mm^3^	G/W ^a^, %	CE ^a^, %
Lithium treated, Mean ± SD	246.3 ± 3.82	179.8 ± 2.75	66.5 ± 2.42	2.73 ± 0.1	0.51 ± 0.06
Control, Mean ± SD	238.5 ± 3.62	156.6 ± 3.61	81.9 ± 0.52	1.91 ± 0.04	0.43 ± 0.09
*P* value	0.143	0.001	0.01	0.0001	-

^a^Abbreviations: CE, consistent estimator; GMV, gray matter volume; G/W, gray/white matter ratio; TCV, total cerebellar volume; WMV, white matter volume

## 5. Discussion

The present study showed that administration of Lithium carbonate 0.1% (w/v) by drinking water in rat for a period of 12 weeks could cause an increase in gray matter volume and a decrease in white matter volume. There were no statistically significant differences in total volume of cerebellum between two groups. It has been shown that the cerebellum is particularly vulnerable to intoxication and poisoning, particularly at cerebellar cortex and Purkinje neurons. In humans, although the most common cause of a toxic lesion of cerebellar circuitry is associated to alcohol ,the cerebellum is also prone to drug exposure (such as anti-neoplastic agents, Lithium salts), drug abuse and addiction, and environmental toxins (15). In line with our results, Sassi et al. using MRI and spectroscopy measured gray and white matter volumes of untreated and Lithium- treated bipolar patients and healthy controls. Their studies showed that total gray matter volumes were significantly increased in Lithium-treated patients compared to untreated patients and healthy individuals. This may reflect neurotrophic effects of Lithium (7). Moore et al. also showed that after 4 weeks of chronic Lithium therapy in bipolar patients, gray matter volume of brain increased significantly probably due to neuroprotective effects of Lithium (8). Studies have exhibited neuroprotective effects of Lithium against cell injuries in cultured cells and also in patients with manic-depressive illness. Lithium has been shown to improve the levels of different neurotransmitters and thus could be responsible for improvement in learning, memory, cognition, and motor functions (16). Neuroprotective effects attributed to the Lithium include protection against such diverse cellular disturbances as increased oxidative stress, apoptosis, inflammation, ethanol-induced neurotoxicity, and mitochondrial and endoplasmic reticulum disruption (17). Lithium supplementation in aluminum treated rats resulted in an appreciable improvement in histoarchitecture of cerebrum and cerebellum. Chronic treatment with Lithium at therapeutic concentrations inhibited glutamate-induced cell death, DNA fragmentation, lipid peroxidation, and protein oxidation in primary-cultured rat cerebral cortical cells (18). Jorda` et al. in their study also detected a number of neuroprotective and antiapoptotic effects of chronic Lithium administration against colchicines. Inhibition of caspase-3 by Lithium may explain the neuroprotective properties of Lithium in neuronal cells (19). Lithium has robustly increased the level of cytoprotective protein bcl-2 in areas of rodent brains and in human neuronal cells (9, 10). In addition to exerting major neuroprotective effects, bcl-2 exhibits antiapoptotic and neurotrophic effects and improves the regeneration of central nervous system axons in mammalian CNS (9, 19) and neurogenesis in some area of rodent brain (10). Chronic Lithium therapy may also increase the number of non-neuronal cells including progenitor cells and glia (10). Other studies have recently provided evidence of Lithium-induced increases in gray matter volumes and N-acetyl- aspartate level. N-acetyl- aspartate is believed to be localized mainly in the dentrites rather than cell body and is a putative marker of neuronal viability and function (8). Increase of gray matter detected by us would be in consent with these studies. On the other hand, Licht et al. using stereological methods in cerebellum and neocortex of rats reported that there were no statistically significant differences between Lithium treated rats with or without haloperidol and control rats after a period of 30 weeks (11, 12). Sassi et al. measured gray matter at right and left anterior and posterior cingulated cortices using MRI. These images revealed a decrease in left anterior cingulated volumes in untreated bipolar patients in comparison with controls, but no statistically significant differences between Lithium-treated patients and controls (13). They concluded that Lithium monotherapy would be capable of preventing atrophy of this important region or reversing the cingulated volume back to normal levels. De Almeida Souza et al., showed that Lithium therapy led to a marked decrease in glycogen content in whole brain. Glycogen metabolism might be implicated in the pathogenesis of bipolar disorder and its diminished synthesis by Lithium can be relevant to treated patients (20). Our study showed that white matter volume in Lithium treated group decreased compared to control group. In cerebellum, in contrast to brain, it is believed that there are no corticocortical projections. Mossy fibers projecting from other parts of the brain probably make up a significant proportion of cerebellar white matter (21). Thus, reduction of white matter volume can be attributed to reduction or atrophy of cerebellar afferent fibers; (i.e. mossy fibers) after a long period of Lithium therapy. Neurologic effects such as movement disorders, ataxia, and other disturbances occurring during prolonged therapy could be arising from this effect. Our previous study showed that there were no significant differences in terms of total volume gray matter and white matter of cerebrum between control and Lithium administrated groups after a period of 12 weeks. It was concluded that Lithium carbonate 0.1% might not affect volumetric parameters and macroscopic structure of cerebrum in rats (14). Niethmer and Ford in a review and case report stated that there were no gross abnormalities in the appearance of patients’ brains. Strikingly, all cases showed similar cerebellar lesions. The cardinal feature was a significant loss of Purkinje cells with sparing of surrounding basket cells. Astrocytosis as well as vacuolization of the white matter and dentate nucleus were observed in some cases (3). Acute Lithium intoxication in rats has been shown to cause widespread vacuolization and spongiform changes in cerebellar white matter while Purkinje cells were spared. Moreover, vacuolization appeared reversible in rats when they were examined 15 days after Lithium injection (3, 6). Efficacy of Lithium treatment may depend on several variables including compound formulation, dosage, and delivery method, and therefore understanding the underlying mechanisms involved in Lithium’s neuroprotective effects will be a paramount next step in these ongoing studies (16). The present study showed that treatment with Lithium carbonate 0.1% (w/v) after a period of 12 weeks can cause an increase in gray matter volume and a decrease in white matter volume. Further studies about the cellular and ultra- structural changes are necessary.
